# The Beginning of a New Volume

**Published:** 1901-01

**Authors:** 


### THE BEGINNING OF A NEW VOLUME.

It is always of interest, in any avocation of life, when the period arrives to close the books of a year and to count the profit or loss in the work upon which any one may have been engaged.

The December number closed with this journal another year of work, a year of anxiety, of toilsome effort to give to the dental profession a journal that would be a true exponent of the best life in dentistry. Its success or failure in this direction has now passed into history, but that it has performed its full share of the labor given to each of us to do may be accepted, and credit be given, at least, for an honest and untiring effort to raise dentistry to a position worthy to rank with the older professions.

The International Dental Journal has now been working under that name for some twelve years, nearly eleven of which have been under the present management. Prior to that time it was known as *The Independent Practitioner* and has now reached its twenty-second volume under both names. During these periods it has been supported by the most earnest men in dentistry. Their sacrifices need no record here, and possibly these may never be known, nor is it material that they should ever be considered.

To establish a journal in the face of an almost overwhelming apathy on the part of dentists to anything in the direction of developing a true professional spirit has required a persistent and courageous effort. The commercial side of dentistry has had the power that capital always gives, and while this journal has been to a remarkable degree successful, it has not and cannot attain to that position its founders hoped for while it must contend with this power, unless it receives the co-operation and continual sympathy of those who desire to see dentistry stand free from all entangling alliances.

It must be clear to all observers that the dental periodical literature of to-day in this country can never reach a true standard of independence while under the control of those not immediately connected with dentistry as a profession. This statement means no reflection on those who are monthly sending out these journals as advertising periodicals. That the majority of the twenty-six thousand dentists of this country absorb their dental pabulum from this source is not to be questioned, with the result that the standard of thought is by no means as high as it should be.

It is not, however, the object of this writing to judge the character of our trade contemporaries, for they must stand or fall on their own merits, but to urge those who feel that dentistry deserves something more in accord with it as a profession, and that those connected with it should make a more pronounced effort to support the only journal founded on a true independent basis. The necessity for an increased subscription list over that of other journals must be clear to the average business mind. It is needed that the journal may be placed on a solid financial foundation; but it is also more imperatively needed in order that it may extend its influence in the direction of a higher professional standard.

To those, therefore, who have already done much, the request is that they do more work in their societies. This is the best direction to labor, and this is the proper time to begin this effort,—at the opening of a new volume.

The International Dental Journal was founded as a medium for independent professional thought. It does not invite controversial papers in which a partisan bias is too plainly apparent, but it offers a place where all the product of the dental mind is welcomed, for in this way only can truth be discovered. The management have no commercial interests that require protection, but they have an ever abiding faith that progress in any direction does not come by sitting idly with folded hands and dumb voices, but that all advance is the result of persistent effort to rouse the apathetic to a clearer perception of their duty to themselves and to the dental profession.

